# Further delineation of complex chromosomal rearrangements in fertile male using multicolor banding

**DOI:** 10.1186/1755-8166-1-17

**Published:** 2008-08-07

**Authors:** Nilüfer Karadeniz, Kristin Mrasek, Anja Weise

**Affiliations:** 1 Ankara Zübeyde Haným Maternity Hospital, Genetic Section, Ankara, Turkey; 2 Institute of Human Genetics and Anthropology, Kollegiengasse 10, D-07743 Jena, Germany

## Abstract

**Background:**

Complex chromosomal rearrangements (CCRs) are defined as structural chromosomal rearrangements with at least three breakpoints and exchange of genetic material between two or more chromosomes. Complex chromosomal translocations are rarely seen in the general population but the frequency of occurrence is anticipated to be much higher due balanced states with no phenotypic presentation. Here, we report a severely mentally retarded fertile male patient in whom further delineation of CCR involving chromosomes 1, 4 and 2 was carried out by using high resolution multicolor banding (MCB) technique. As a FISH based novel chromosome banding approach, high resolution MCB allows for the differentiation of chromosome region specific areas at band and subband levels.

**Results:**

Cytogenetic studies using high resolution banding of the proband necessitated further delineation of the breakpoints because of their uncertainty: 46,XY,t(1;4;2)(p21~31;q31.3;q31). After using high resolution MCB based on microdissection derived region-specific libraries, the exact nature of chromosomal rearrangements for chromosomes 1, 2 and 4 were revealed and these breakpoints were located on 1p31.1, 1q24.3 and 4q31.3 giving rise to a balanced situation.

**Conclusion:**

Further delineations are certainly required to provide detailed information about the relationship between balanced CCRs and their phenotypes in order to offer proper counseling to the families concerned. Carriers must be investigated with high resolution banding and molecular cytogenetic techniques to determine the exact locations of the breakpoints. High resolution MCB is an alternative and an efficient method to other FISH based chromosome banding techniques and can serve in clarifying the nature of CCR.

## Background

Structural chromosomal abnormalities are estimated to occur in around 0.5% of newborn infants, using moderate level of resolution in conventional cytogenetic analysis [1]. Complex chromosomal rearrangements (CCRs) are defined as structural chromosomal rearrangements with at least three breakpoints and exchange of genetic material between two or more chromosomes. It is therefore not surprising to see CCR rarely in constitutional karyotypes. Moreover, some CCRs cannot be interpreted with standard cytogenetic methods at all [2]. Complex chromosomal rearrangements are extremely rare but are often associated with mental retardation, congenital abnormalities, recurrent abortions and infertility [3]. More than 130 constitutional CCRs have been documented so far [4]. 12 of these were related with fertile men including the case we present [5]. Providing genetic counseling for CCRs is very important and this can be offered before or after pregnancy as well as at the time of prenatal diagnosis [6].

Since the introduction of fluorescence in situ hybridization (FISH) techniques using whole chromosome painting probes [7] in human cytogenetics, progress has been achieved concerning the ability to characterize chromosomal subregions by molecular cytogenetic methods. Recently, high resolution MCB technique was developed [8] making it possible to identify different chromosome region specific areas at band and subband levels.

Here we report a fertile male with mental retardation carrying balanced complex chromosomal rearrangements, involving chromosomes 1, 4 and 2. We also provide advice for genetic counseling of the fertile CCR carrier by discussing the possible mechanisms underlying the origin of CCR.

## Results

**Figure 1:**
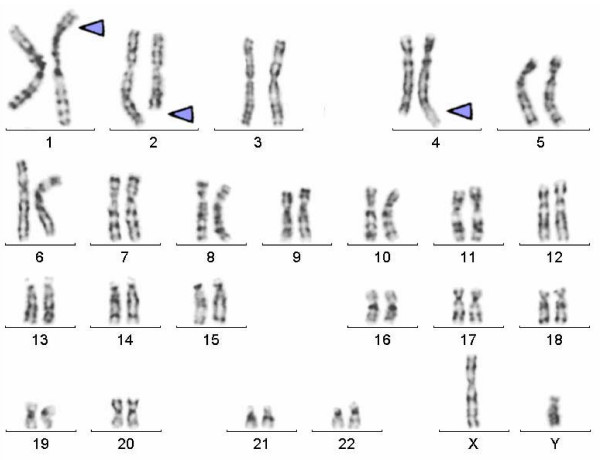
**shows image of the GTG banded metaphase**. Image belongs to GTG banded metaphases from the father. Arrow indicates the breakpoints on each chromosome.

**Figure 2:**
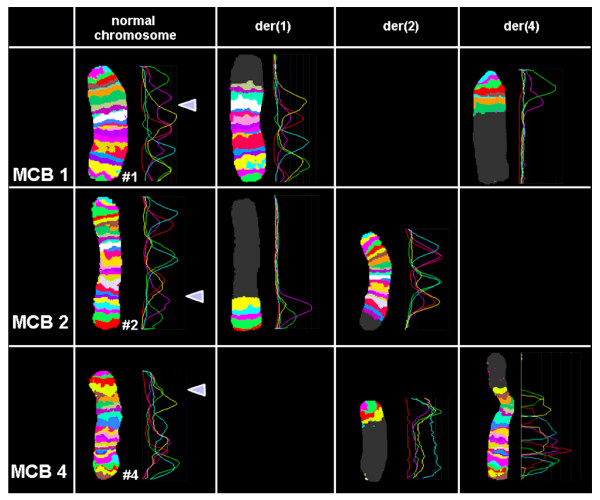
**shows MCB pattern of the cytogenetic result**. It shows multicolor banding (MCB) applying probe-sets for chromosomes 1, 2 and 4 characterized the breakpoints as 1p31.1, 2q24.3 and 4q31.3. The corresponding results are shown here. For each chromosome depicted the MCB-pseudo-colors as well as the underlying fluorescence profiles are shown (for details of MCB evaluation see [22]. The first of the 5 columns shows which probe set was used (MCB 1, 2 or 4). The second column shows the normal chromosomes #1, #2 and #4. The arrowhead shows the breakpoint as present in the derivative sister-chromosomes. Third to fifth columns show the derivative chromosomes (der) 1, 2 and 4. MCB 1 stains parts of der(1) and der(4), MCB 2 parts of der(1) and der(2) and MCB 4 parts of der(2) and der(4). Parts not stained by the corresponding MCB probe-sets are pseudo-colored in gray. Overall, the complex chromosomal rearrangement was balanced, according to molecular cytogenetics.

Banding cytogenetic revealed a normal karyotype for the wife and a complex rearranged one for the spouse. A CCR involving chromosomes 1, 2 and 4 was detected and his karyotype was characterized as 46, XY, t (1; 4; 2) (p21~31; q31.3; q31) (see Fig 1). After performing FISH by using MCB (see Fig 2), the breakpoints were localized to 1p31.1, 2q24.3 and 4q31.3.

## Discussion

As existing difficulty of precise definition of CCR using standard cytogenetic methods [2], detection of CCR in the chromosomes of a patient causes anxiety for patient and clinician, especially when it is balanced that can lead to genetic imbalance [9]. Because precise identification of all the chromosomes involved in a CCR is the prerequisite to every appropriate genetic counseling. By combining high resolution techniques of chromosome banding with FISH we have an essential tool to determine whether a complex abnormal karyotype is apparent or not, this is especially important for prenatal diagnosis [10].

These rearrangements are usually ascertained by routine chromosome analysis of a child with mental retardation and congenital abnormalities [11], recurrent abortions in female [12], and infertility in man [13]. Although most carriers of balanced translocations are phenotypically normal, in a small proportion (~6%) of these phenotypic abnormalities are reported [9,14]. Madan et al [1997] analyzed 60 cases with balanced CCRs [6]. They found that for female CCR carriers the risk of abortions and abnormal livebirths is 52.6% and for male carriers the risk of abortion and abnormal livebirths is 60%, with a combined frequency of 53.7%. While liveborn infants possessing normal chromosomes have incidences of 31.6%, liveborn infants carrying balanced chromosomes have incidences of 50% [6]. If chromosomal rearrangement is detected in a phenotypically normal individual, then this rearrangement is generally assumed to be truly balanced. These often represent familial cases. If, however, a chromosomal rearrangement is detected in a phenotypically abnormal individual; then usually a submicroscopic imbalance or other genetic defects exist. This situation often represents de novo cases. The incidence of live born infants with unbalanced chromosomes and variable degrees of phenotypic abnormalities is 18.4% [6,15]. An abnormal phenotype with apparently balanced rearrangements may be the result of chromosomal breakage disrupting a gene leading to abnormal gene expression or the presence of a submicroscopic deletion or duplication [9]. The change of location and/or orientation of translocated genes can also influence the activity of regulatory sequences co-operating with the breakpoint flanking translocated genes [16]. Recently Goumy et al [2006] described a boy with mild developmental delay and psychotic disorder. He had balanced complex rearrangements but no molecular abnormalities were detected by using FISH with whole chromosome painting (WCP), comparative genomic hybridization (CGH) and array-CGH [4]. The results of array CGH belong to De Gregori et al. [2007] showed that 16 of 18 patients had imbalances while all cases had been interpreted as balanced by conventional cytogenetics. 11 of 16 CCRs associated with deletion. The phenotypic abnormalities of apparently balanced de novo CCRs are mainly due to cryptic deletions. There was no association between the severity of the pathology and the number of deletions or their sizes [17].

Moreover, the exact cytogenetic mechanisms underlying the origin of CCRs are unclear. A major catastrophe within the gamete (spermatogenesis) appears a vague yet plausible patognomonic mechanism for CCRs [15]. Simple three-way translocations are predicted to form hexavalents at meiosis. By focusing solely on symmetric segregation (3:3), up to 20 possible gametic combinations could be devised among which only two were balanced. The number of unbalanced gametes increased significantly together with the possibility of asymmetric segregation and recombination during meiosis [5]. Lespinasse et al. [2003] analyzed the localization of 90 chromosome breakpoints in 24 CCRs delineating random involvements of specific chromosomes in CCRs. However, they observed a non-random distribution of specific breakpoints at 1q25, 4q13, 6q27, 7p14, 9q12, 11p11, 12q21, 13q31 and 18q21 [13]. Recently De Gregori et al. [2007] screened 59 balanced translocations including CCRs by using array comparative genome hybridization and 18 of these were found to be de novo balanced complex translocations. At the 22 breakpoints identified using a specific customized array, they could not find any specific DNA sequences. Thus, they were unable to determine the mechanisms underlying the concurrent breakage of several chromosomes with losses of parts of the broken portions and their random assortment. Considering that all the men fathering children with unbalanced translocation or CCRs were fertile, they came up with the following hypothesis: during spermatogenesis some cells escape the mechanism responsible for correct crossing-over; these undergo chaotic breaks resulting in the reunion of several chromosomes and thereby exposing the broken portions to exonuclease degradation [17].

The couple we present in here has only one living child out of four pregnancies. Their male child does not have any clinical abnormalities or any developmental delay. But we do not have any objective findings to confirm whether this child bears any chromosomal abnormalities. Even if the detected CCR looks balanced with MCB, the carrier of this CCR has severe mental deficiency. Several studies reported apparently balanced chromosomal rearrangements to be associated with significant risks of mental retardation and malformation [11,16]. Based on a review of apparently balanced translocations, Warburton [1982] concluded that the presence of a de novo apparently balanced translocation is associated with an increased risk of mental retardation with an odds ratio of 6.0–7.0 [18]. Moreover, the vast majority of male carriers show reduced fertility [19,20]. Disturbances in spermatogenesis as well as pre- and post implantation losses are discussed as reasons for this phenomenon [6]. Zahed et al. [1998] suggested that the scarcity of the number of transmitting males with CCRs is usually attributed to either a lower risk of producing abnormal progeny therefore, a lower probability of ascertainment, or to infertility attributed to problems in chromosome pairing at spermatogenesis [21]. There are only few reports on fertile male CCR carriers referred for cytogenetic evaluation due to spontaneous abortions of spouses or due to abnormal offsprings, which were well reviewed by Grasshoff et al. [2003] as presented here [5].

Identification of submicroscopic aberrations (below 3 Mb) and more detailed molecular profiling of the rearrangements require precise mapping of the breakpoints with other methods such as florescence in situ hybridization (FISH) with locus-specific probes or array CGH [9]. Since the establishment of FISH technique in human cytogenetic, much progress has been achieved concerning the ability to characterize chromosomal subregions by molecular cytogenetic methods. Recently, high resolution multicolor banding (MCB) technique was developed. By producing changing florescence intensity ratios along the chromosomes, MCB approach allows the differentiation of chromosome region specific areas at the band and subband levels and is based on region specific microdissection libraries [8]. MCB technique is a high resolution alternative suited to clarify the changes appearing in complex chromosomal rearrangements [22]. We used MCB techniques for certain determination of the breakpoints on each chromosome and the detection of possible deletions. According to MCB results the proband has balanced complex chromosomal translocations. Liehr et al [2002] suggested that the MCB-technique is a high resolution alternative to other FISH based chromosome banding approaches and it suits to clarify the changes appearing in CCRs [22].

## Conclusion

Further delineations are certainly required to provide more information about the relationships between balanced CCRs and their phenotypes. Determination of certain breakpoints is also important for counseling the patients. With these, correct prenatal diagnosis and efficient genetic counseling can be possible for the carriers of CCR. The couples with CCR should be also informed about the possible outcomes of the progeny and the fact that exact risk of malformation is still unknown and that phenotypically normal child can still have a high risk of reproductive problems. The carriers must be investigated with high resolution banding and molecular cytogenetic techniques in order to see whether the CCR is truly balanced or not and if balanced where these breakpoints are located. Finally, high resolution MCB techniques by themselves can be used as alternative methods to determine exact locations of the breakpoints.

## Materials and methods

### Clinical case report

A couple was referred to us following three pregnancy losses out of four pregnancies. The mother was 26 years old and had one living child from her third pregnancy. The first one was lost at the first trimester, the second one was aborted at the third trimester due to fetal abnormality, the third one was finally born 4 years ago as a healthy male child, and the fourth one was lost at the first trimester again. The father was 33 years old, he had mental deficit since birth while his spermiogram was normal. There were no functional motor deficits apart from the severe mental retardation he suffered necessitating continuous support. The examination of their male child did not reveal any clinical evidence about any abnormality. The parents did not give consent to further evaluate their living child with chromosomal analysis.

### Banding cytogenetics

Cytogenetic investigations from the couple were performed on peripheral blood samples using a high resolution technique after cell culture synchronization and BrdU incorporation [23].

### Molecular cytogenetics

High resolution multicolor banding (MCB) based on microdissection derived region-specific libraries for chromosomes 1, 2 and 4 was carried out to further delineate the nature of chromosomal rearrangements as described before [22]. Each of the 20 metaphase spreads were analyzed by using a fluorescence microscope (Axioplan 2 mot, Zeiss) equipped with appropriate filter sets to discriminate between a maximum of five fluorochromes and the counterstain DAPI (Diaminophenylindol). Image capturing and processing were carried out using an ISIS mFISH imaging system (MetaSystems, Altlussheim, Germany) for the evaluation of MCB.

## List of abberations

CCRs: comples chromosomal rearrengements; CGH: comparative genomic hybridization; FISH: fluoresence *in situ *hybridization; MCB: multi colour banding; WCP: whole chromosome painting.

## Competing interests

The authors declare that they have no competing interests.

## Authors' contributions

NK evaluated the family with examination, counseling and cytogenetically, and prepared the revised MS. KM and AW did the molecular cytogenetic analysis and interpretation of the MCB results. All authors' read and approved the final manuscript.
